# aPKC in neuronal differentiation, maturation and function

**DOI:** 10.1042/NS20190019

**Published:** 2019-09-23

**Authors:** Sophie M. Hapak, Carla V. Rothlin, Sourav Ghosh

**Affiliations:** 1 Department of Medicine, School of Medicine, University of Minnesota, 401 East River Parkway, Minneapolis, MN 55455, U.S.A.; 2 Department of Immunobiology, School of Medicine, Yale University, 300 Cedar Street, New Haven, CT 06520, U.S.A.; 3 Department of Pharmacology, School of Medicine, Yale University, 333 Cedar Street, New Haven, CT 06520, U.S.A.; 4 Department of Neurology, School of Medicine, Yale University, 300 Cedar Street, New Haven, CT 06520, U.S.A.

**Keywords:** learning and memory, neurodevelopment, neuronal differentiation

## Abstract

The atypical Protein Kinase Cs (aPKCs)—PRKCI, PRKCZ and PKMζ—form a subfamily within the Protein Kinase C (PKC) family. These kinases are expressed in the nervous system, including during its development and in adulthood. One of the aPKCs, PKMζ, appears to be restricted to the nervous system. aPKCs are known to play a role in a variety of cellular responses such as proliferation, differentiation, polarity, migration, survival and key metabolic functions such as glucose uptake, that are critical for nervous system development and function. Therefore, these kinases have garnered a lot of interest in terms of their functional role in the nervous system. Here we review the expression and function of aPKCs in neural development and in neuronal maturation and function. Despite seemingly paradoxical findings with genetic deletion *versus* gene silencing approaches, we posit that aPKCs are likely candidates for regulating many important neurodevelopmental and neuronal functions, and may be associated with a number of human neuropsychiatric diseases.

Atypical Protein Kinase Cs (aPKCs) are a group of protein kinases with an evolutionarily conserved role in regulating cell polarity—a feature central to essentially all aspects of neuronal biology. This includes neural stem cell or neuronal progenitor cell proliferation, differentiation, directional migration, asymmetric cellular domain formation and vectorial functions, such as neurotransmitter release. Therefore, aPKCs can rationally be expected to play critical roles in neuronal development and function. Indeed, multiple studies have supported a crucial role of aPKCs in neuronal development and function. Notwithstanding, this field of study has occasionally been mired by controversies due to contradictory outcomes of other, independent studies. Therefore, many aspects of aPKC function in neurobiology still remain somewhat ambiguous. We attempt to provide some background on the biochemical action of aPKCs, their expression profile in the nervous system and summarize the panoply of functions (or the lack thereof) ascribed to these kinases during nervous system development and activity.

## aPKC: discovery, classification and transcripts

Eukaryotic protein kinases characterized by a conserved catalytic domain are primarily categorized into seven major groups, including the AGC (protein kinase A, protein kinase G and protein kinase C) kinase group. PKCs form a family within this group. PKCs can be further classified into three subfamilies—classical/conventional PKCs, novel PKCs and aPKCs. The first aPKC was identified by Ono et al. [1,2] in Yasutomi Nishizuka’s laboratory from a rat brain complementary DNA (cDNA) library using classical/conventional PKC probes and homology cloning. When the full-length clone was expressed in COS-7 (CV-1[simian] origin carrying SV40 genes) cells, its basal enzymatic activity was independent of calcium ion (Ca^2+^), phospholipid or diacylglycerol (DAG) [2]. However, the addition of the phospholipid phosphatidylserine, but not DAG or phorbol ester (phorbol 12-myristate 13-acetate), enhanced its activity [2]. Thus, this protein was classified within the PKC family due to its kinase domain amino acid identity, but as a distinct subgroup as its requirements for its kinase activity were distinct from the Ca^2+^- and DAG-dependent classical/conventional PKCs (α, βI, βII and γ) and Ca^2+^-independent but DAG-dependent novel PKCs (δ, ε, η and θ) [3,4]. PKCs bind Ca^2+^ through their C2 domain and DAG/phorbol esters through their two C1 domains. The C2 domain of novel PKCs does not bind Ca^2+^, while aPKC lack this domain entirely [3]. Additionally, aPKCs contain only a single, atypical C1 domain [3].

**Figure 1:**
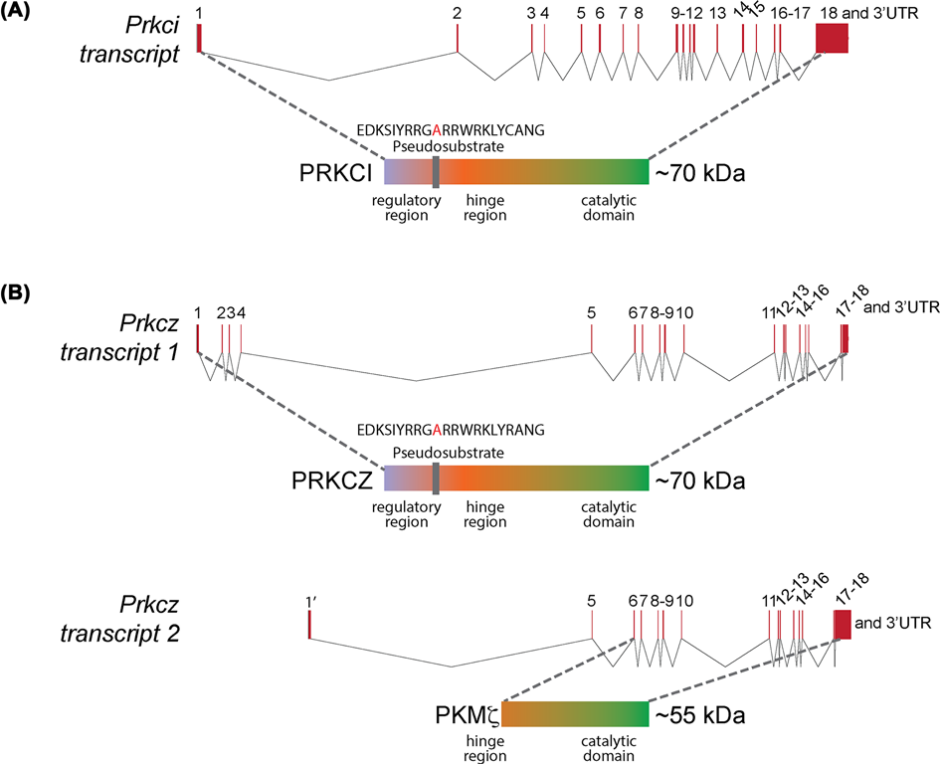
Mammalian aPKCs Schematics of aPKC transcripts and proteins encoded by (**A**) *Prkci* and (**B**) *Prkcz* (not drawn to scale). *Prkci* codes for a single full-length transcript and a ∼70 kDa protein with regulatory, hinge and catalytic domains. The exons (in red boxes) are numbered and the splice events are shown in thin black lines. Thus, Exon 1 is spliced to Exon 2, which in turn is spliced to Exon 3 etc*.* The translation start-site (ATG) is within Exon 1. *Prkcz* codes for at least two transcripts in mice and more in humans. The full-length transcript encodes a ∼70 kDa protein with regulatory, hinge and catalytic domains that share ∼72% amino acid identity with PRKCI. The exons (in red boxes) are numbered and the splice events are shown in thin black lines. Thus, Exon 1 is spliced to Exon 2, which in turn is spliced to Exon 3 etc. The translation start-site (ATG) is within Exon 1. PKMζ is coded by a shorter transcript from an alternative transcription start site (GCCGTGTTTTAGC…) that skips Exon 1 through 4 but contains a unique exon (Exon 1′ located between Exon 4 and Exon 5 in the gene). Exon 1′ is not present in the full-length *Prkcz* transcript. PKMζ protein lacks the entire regulatory and a part of the hinge region of PRKCZ, but contains the entire catalytic domain. The exons (in red boxes) are numbered and the splice events are shown in thin black lines. Thus, Exon 1′ is spliced to Exon 5, which in turn is spliced to Exon 6 etc*.* The translational start site for PKMζ (ATG) is in Exon 6. Figure is created using the webtool Exon-Intron Graphic Maker (wormweb.org).

The newly termed aPKC cDNA was termed as PKCζ. Another aPKC was identified first in rat and hamster cell lines, and subsequently in human kidney cDNA library by Selbie et al. in 1993 [5]. The product of this gene, designated as PKCι, exhibited 72% amino acid sequence identity to PKCζ. Akimoto et al. [6] independently cloned this gene from mouse postnatal day 19 (P19) embryonal carcinoma cells in 1994, and named it PKCλ. PKCι and PKCλ are the same gene and now identified as *Prkci* across all species. Thus, in vertebrates, there are two aPKC genes—*Prkci* or aPKC ι/λ and *Prkcz* or aPKCζ (Figure 1). In this review, we will use the nomenclature PRKCI and PRKCZ for the full-length proteins of *Prkci* and *Prkcz. Prkcz* not only codes for PRKCZ but also at least one additional transcript that skips the first four exons and instead starts in an alternative exon (1′) not represented in the full-length transcript [7] (Figure 1). We will use the term PKMζ to refer to this protein.

Due to skipping the first four exons during transcription and translation actually starting from exon six, PKMζ lacks the N-terminal regulatory domain contained in full-length aPKC proteins. This regulatory domain contains the inhibitory pseudosubstrate motif characteristic of the PKC family, in addition to a PB1 (Phox/Bem1) domain [8]—the binding site for the scaffolding proteins including partitioning defective (PARD) 6 (PARD6) and p62/sequestosome-1 (p62/SQSTM) present in PRKCI and PRKCZ. PARD6 is a binding partner of aPKCs and is important for cell polarity [9]. p62/SQSTM is a component of NF-kB signaling and an important regulator of autophagy [10–12]. PKMζ, thus, lacks the inhibitory pseudosubstrate motif and is constitutively active, and is also incapable of binding PARD6 and other proteins that interact through this PB1 domain.

aPKC appears to evolve in pre-metazoans. Although the choanoflagellate *Monosiga brevicolis* lacks aPKCs, and indeed the entire AGC kinase family [13], an ortholog of vertebrate aPKCs is present in the filasterean *Capsaspora owczarzaki* [14,15]. In the desmosponge *Amphimedon queenslandica*, an early-branching metazoan lineage, as well as true eumetazoans, aPKC is conserved [16]. The soil-inhabiting nematode *Caenorhabditis elegans* (*C. elegans*), the fruitfly (*Drosophila melanogaster*) and the tunicate *Ciona intestinalis* have a single aPKC termed as *Drosophila* aPKC (DaPKC), PKC3 and Ci-aPKC, respectively [17–20]. Vertebrate aPKCs may be a result of the large-scale DNA duplications in an early chordate [21]. Interestingly, the alternative translation start-site for PKMζ within *Prkcz* is conserved in all vertebrates including Tetraodontidae such as pufferfish (*Fugu rubripes*) [22]. In contrast, this methionine is absent from *Prkci*, as well as in aPKC in the deuterostomes, indicating that the translational start site for PKMζ arose after the genome duplication event that generated the paralogs *Prkci* and *Prkcz* [22].

It is important to point out that only few studies correctly discriminate between PRKCI *versus* PRKCZ *versus* PKMζ when investigating mammalian aPKC functions. Reasons for the lack of rigor may include perceived functional redundancy between the aPKCs (stemming from their ∼72% amino acid identity overall and ∼86% amino acid identity in the kinase domain) or reliance on improper reagents. Unfortunately, this has generated considerable confusion in the field in terms of identity and function of the individual aPKCs. Many studies report PRKCZ functions in cells or tissues where this particular aPKC is not expressed (*please see below*). There is an urgent need for the use of properly validated reagents when studying aPKCs. Incorrect identity or the lack of specific identity in many studies have likely contributed to paradoxical results and controversies regarding aPKC function in the nervous system.

## aPKC activation

**Figure 2:**
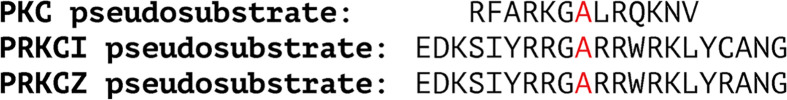
PSs of classical/conventional PKC and aPKCs PSs of PRKCI and PRKCZ are aligned with R^19^ FARKGALRQKNV^31^ PS of classical/conventional PKCs. The PSs are enriched in R/K positively charged amino acids at neutral pH.

Although kinase-independent functions have been described for aPKC [23,24], most studies ascribe aPKC function to its kinase activity. However, the rate of *in vitro* enzymatic activity of aPKCs is slow compared with that of other PKCs—the catalytic activity of PRKCZ was calculated to be 5 mol of phosphate/min/mol of kinase, while that of PKC βII was 200 mol of phosphate/min/mol of kinase [25–27], and the mechanism of activation of its enzymatic kinase activity is still under scrutiny. The full-length aPKCs, PRKCI and PRKCZ, contain a pseudosubstrate sequence (PS) which keeps them inactive but poised for activation (Figure 2). Agonist-dependent activation of aPKC may occur through the displacement of the pseudosubstrate. Agonist-dependent activation of full-length aPKC in the nervous system remains mostly undescribed, although a few studies have described WNT (portmanteau from Wingless and Int)- or IGF-1 (insulin-like growth factor 1)-dependent full-length aPKC activation during neuronal polarization [28–30] (reviewed in [31]). As described earlier, PKMζ lacks PS and is constitutively active [32,33]. The major step in the regulation of PKMζ appears to be its synthesis, which is under the control of many kinases implicated in long-term potentiation (LTP)—a persistent increase in synaptic strength following high-frequency stimulation. These include PI3-K, MAPK (mitogen-activated protein kinase), CaMKII (Ca^2+^/Calmodulin-dependent protein kinase) and PKA [32].

Apart from agonist-dependent induction in its enzymatic activity, many other factors are proposed to regulate aPKC kinase activity. While aPKCs are Ca^2+^ or DAG-insensitive, many studies have described a lipid-dependence for the kinase activity of the full-length aPKCs [2]. For example, ceramide, phosphatidylserine and more recently, sphingosine 1-phosphate (S1P), have been described to activate the full-length aPKCs [34–39]. A study by Lopez-Garcia et al. [40] reported that PS interacted with the catalytic domain and PS was not only responsible for PRKCZ activation by lipids but also regulated the stability of this kinase. Unlike the full-length aPKCs, PKMζ kinase activity is independent of lipids [41].

**Figure 3:**
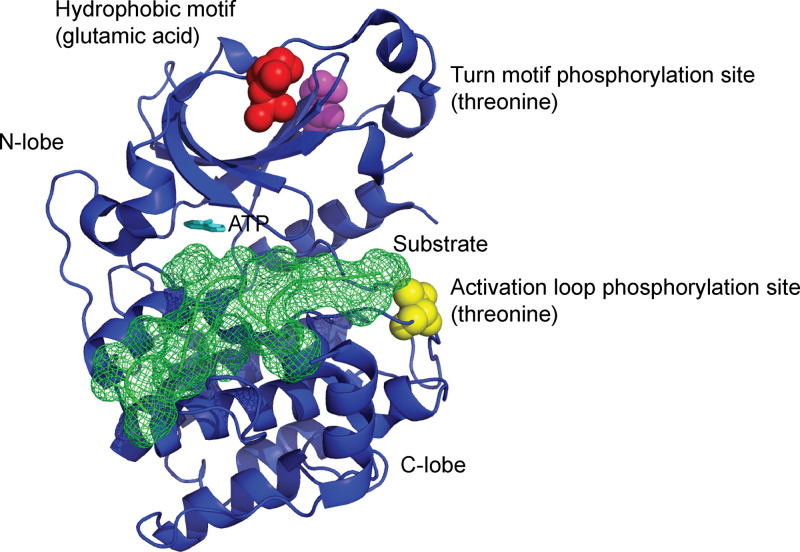
The priming phosphorylations of aPKCs The hydrophobic motif characteristic of PKCs contains a phospho-acceptor residue. In aPKCs, this site contains a negatively charged glutamic acid residue (red balls). Notwithstanding, aPKCs share phospho-acceptor residues in the turn-motif (purple balls) and the activation loop (yellow balls) that are phosphorylated by mTORC2 and PDK1, respectively. These phosphorylations appear to be necessary for aPKC activity. The substrate binding site (green network) and ATP is also shown.

The classical/conventional and novel PKCs require priming phosphorylations at the activation, hydrophobic and turn-motifs to be rendered ready for activation. aPKCs can also be phosphorylated in their activation- and turn-motifs, but the hydrophobic motif already carries a charged residue and does not require further phosphorylation [3] (Figure 3). aPKC phosphorylation at the activation- or turn-motif has been commonly interpreted as indicative of activation [3]. This remains to be fully validated. The recent observation that aPKC activation loop phosphorylation was not agonist-induced and its activity, as monitored by a genetically encoded fluorescent reporter, was constitutive following insulin stimulation supports the notion of co-translational priming phosphorylations [26]. Indeed, the activation loop phosphorylation in PKMζ is likely to be co-translational. While PKMζ is constitutively active, it has been reported that its activation still requires the priming phosphorylation of its activation motif [42]. PKMζ isolated from the hippocampus is maximally phosphorylated at its activation loop and the relative amounts of phosphorylated to total PKMζ was found to be unaffected by LTP [32]. Furthermore, PKMζ and PDK1 (pyruvate dehydrogenase kinase), the kinase responsible for the activation loop phosphorylation, were found to be present in a complex with each other [32]. In contrast, the relative amount of activation loop phosphorylation of PRKCI to the total amount of this protein was shown to increase after LTP [32].

In summary, the sequence from synthesis to activation of the full-length aPKCs may be (i) regulation of new protein synthesis, (ii) co-translational priming phosphorylation, followed by (iii) autoinhibition through PS and (iv) induced activation through relief of autoinhibition following agonist-driven signaling, lipid interaction or association with activating/dissociation from inhibitory scaffolds. For the shorter PKMζ, it is expected that the co-translational priming phosphorylations will be necessary and sufficient for activation as it lacks post-translational autoinhibition through PS. Evidence also exists for a positive feedback loop wherein PKMζ can directly regulate its own translation. Pin1 can interact with the translation initiation and elongation factors eIF4E (eukaryotic translation initiation factor 4E) and 4EBP1/2 (eIF4E-binding protein 1/2) and functions as a translational repressor [43]. PKMζ phosphorylates and inactivates Pin1 [43]. This in turn, leads to increased protein amounts of PRKCZ and PKMζ [43]. Therefore, at least PKMζ activation, and perhaps also full-length aPKCs activation, may be linked to new protein synthesis and enhanced co-translational priming phosphorylation.

Other activation mechanisms for aPKCs have also been described. One such activation mechanism for full-length aPKCs involves proteolytic cleavage. Caspase and calpain cleavage between the regulatory and kinase domains have been reported to activate PRKCZ [44–48]. Cleavage is the primary mechanism for generation of PKMζ in invertebrates. As mentioned earlier, PKMζ in vertebrates is directly translated lacking the regulatory domain and PKMζ has enhanced basal kinase activity in comparison with full-length aPKCs [25].

Yet another important mechanism of activation of full-length aPKCs is through their interactions with their scaffolds [25]. Reports on whether specific interactions, such as the interaction with PARD6, are activating or inhibitory have been contradictory [25,49–52]. Nonetheless, scaffold-interactions appear critical for regulating aPKC activity either by direct activation or by relief of repression. PKMζ lacks the domain required for PARD6 interaction, but retains the PARD3 interaction [53].

## aPKC inhibitors

Two approaches are commonly used to inhibit aPKCs—molecular/genetic and pharmacological. As far as molecular/genetic approach is concerned, a mutation that substitutes the catalytic lysine of aPKCs for arginine or tryptophan is frequently used and described as dominant-negative. Presumably, these mutants bind substrates and then fail to phosphorylate them, which effectively sequesters substrates from other active aPKC molecules. However, at least one paper indicates that PRKCI kinase activity is preserved after the lysine is changed to arginine, although it is lost when the substitution is to tryptophan [54]. Therefore, consensus kinase-inactive mutations should be further investigated for each of the three aPKCs.

Many pharmacological inhibitors of aPKCs have been described (Table 1). The most commonly used specific inhibitor of PRKCZ and PKMζ is a membrane-permeable peptide based on the PS of PRKCZ (pseudosubstrate inhibitor). This PS is essentially an arginine/lysine-rich positively charged sequence, not dissimilar to a general PKC PS [55] (Figure 2). The aPKC pseudosubstrate inhibitor, termed as ZIP (ζ-inhibiting peptide) has been historically used in LTP and memory studies. However, ZIP not only blocks PRKCZ but also PRKCI [41]. In fact, ZIP also blocks the enzymatic activity of PKMs derived from classical/conventional PKC, at least in the sea slug (*Aplysia californica*) [47]. Typically, ZIP is also myristoylated for membrane targeting. There is some controversy over the cell penetration and effectiveness of ZIP—while Wu-Zhang et al. [56,57] described that ZIP failed to inhibit PKMζ in cultured cells or in rat hippocampal slices even at 1 μM, despite its effectiveness in *in vitro* kinase assays, other reports validated this reagent and indicated that the overexpression of PKMζ, such as in the Wu-Zhang study, can reduce or eliminate the effects of ZIP [58].

**Table 1:** Commonly used inhibitors of aPKCs

Inhibitor	IC_50_*	Specificity	Reference
ZIP	∼ 1 μM (PKMζ)^†^	All aPKCs, other PKCs, other kinases	Ling et al. [59]
			Lisman [133]
			Wu-Zhang et al. [57]
			Yao et al. [58]
			Farah et al. [47]
Chelerythrine	>40 μM (PRKCZ)	Reported to be ∼1 μM^†^ (for PKMζ)	Thompson and Fields [61]
Pfizer 01	∼22 nM (PRKCZ)	∼7x for PRKCZ over PRKCI	Trujillo et al. [63]
Pfizer 03	∼2.26 nM (PRKCZ)	n/a	Trujillo et al. [63]
Pfizer 09 (PZ09)	∼5.18 nM (PRKCZ)	∼10x for PRKCZ over PRKCI	Trujillo et al. [63]
CRT0066854	∼0.132 μM (PRKCI)	∼4.7x for PRKCI	Kjær et al. [70]
	∼0.623 μM (PRKCZ)		
CRT0066390	∼2.5 μM (PRKCI)	∼2.8x for PRKCI	Kjær et al. [70]
	∼7.1 μM (PRKCZ)		
CRT0329868	∼8.4 nM (PRKCI)	n/a	Mirza et al. [71]
2-amino-3-carboxy-4-phenylthiophenes			Titchenell et al. [65]
6	∼6 μM (PRKCZ)	∼1.6x for PRKCZ over PRKCI	
10	∼6 μM (PRKCZ)	n/a	
14	∼5 μM (PRKCZ)	∼2.2x to 1.6x for PRKCI over PRKCZ	
29	∼2 μM (PRKCZ)	n/a	
30	∼1 μM (PRKCZ)	Approx. equivalent	
32	∼2 μM (PRKCZ)	∼2x for PRKCI over PRKCz	
33	∼4 μM (PRKCZ)	n/a	
PS171	<50 μM (PRKCZ)	∼2.5x for PRKCZ over PRKCI	Lopez-Garcia et al. [40]
PS168	<50 μM (PRKCZ)	∼5x for PRKCZ over PRKCI	Lopez-Garcia et al. [40]
4-benzimidazolyl-3-phenylbutanoic acid analog series			Fröhner et al. [72]
1d	∼33 μM (PRKCZ)		
1n	∼38 μM (PRKCZ)		
1o	∼55 μM (PRKCZ)		
1p	∼39 μM (PRKCZ)		
1q	∼18 μM (PRKCZ)		
1s	∼43 μM (PRKCZ)		
1w	∼23 μM (PRKCZ)		
1x	∼33 μM (PRKCZ)	Most PRKCZ selective (no effect on PRKCI)	
1y*^c^*	∼25 μM (PRKCZ)		
1y-e1	∼20 μM (PRKCZ)		
1y-e2	∼37 μM (PRKCZ)		
ICA-1	∼0.1 μM (PRKCI)	Does not inhibit PRKCZ at low conc.	Pillai et al. [67]
[4-(5-amino-4-carbamoylimidazol-1-yl)-2,3-dihydroxycyclopentyl] methyl dihydrogen phosphate			
ACPD	∼2.5 μM (PRKCZ/I)^†^	Approx. equivalent	Ratnayake et al. [66]
2-acetyl-1,3-cyclopentanedione			
DNDA	∼2.5 μM (PRKCZ/I)^†^	Approx. equivalent	Ratnayake et al. [66]
3,4-diaminonaphthalene-2,7-disulfonic acid			

List of compounds described as inhibitors of aPKCs, the concentration that inhibits 50 percent enzymatic activity of the kinase (IC_50_), respective specificities and associated references are listed. Since this list was compiled from several studies, each using different conditions, the IC_50_ are only to be used as approximation and not directly compared with each other. While most IC_50_s were derived from *in vitro* assays, those indicated by ^†^ are from *in vivo* studies.

* Kinase assays were performed at various ATP concentrations and should not be compared across studies. ^†^*In vivo* IC_50_.

Another commonly used aPKC inhibitor is chelerythrine [59]. This is an active-site PKC inhibitor [60,61], that, when used at low concentrations selectively blocked PKMζ over PKCα, PKCε and CaMKII [59]. Surprisingly, in a comprehensive analysis of kinase-specificities of commercially available kinase inhibitors, Davies et al. [62] indicated that chelerythrine did not inhibit any of the 34 kinases tested including PKCα and PKCδ, even at 10 μM concentration of the inhibitor (aPKCs were not included in the kinase panel in the Davies et al. study). The Wu-Zhang et al. study [57] demonstrated that chelerythrine, similar to ZIP, failed to inhibit PKMζ in cells and brain slices, although the concentration of overexpressed PKMζ used in this study has been questioned [58]. The specificity of chelerythrine has been questioned by Wu-Zhang et al. ([57] and references therein).

A series of 2-(6-Phenyl-1H-indazol-3-yl)-1H-benzo[d]imidazole compounds were designed to inhibit aPKCs [63]. One of the compounds, designated PZ09, has been validated to inhibit aPKCs in cell cultures [4,26,39,52,64]. While at higher concentrations (10 μM) PZ09 can inhibit PKA and p70S6K, 5 μM did not inhibit PKCα in cells [25]. However, PZ09 also inhibits PDK1, the kinase that phosphorylates the activation loop site in aPKCs [25,63].

Many other active-site aPKC inhibitors have been described. Another series of 2-amino-3-carboxy-4-phenylthiophenes have also been described as inhibitors of PRKCZ and PRKCI in cell-based functional assays [65]. Another two aPKC inhibitors, 2-acetyl-1,3-cyclopentanedione and 3,4-diaminonaphthalene-2,7-disulfonic acid, were described by Ratnayake et al. [66] from the Avecedo-Duncan group. These molecules were also effective in cell-based functional assays [66]. The same group also characterized a PRKCI selective inhibitor 5-amino-1-(1R,2S,3S,4R)-2,3-dihydroxy-4-methylcyclopentyl)-1H-imidazole-4-carboxamide [67,68]. This compound has been tested in murine models [69]. Importantly, this inhibitor does not inhibit PRKCZ at concentrations up to 5 μM [67]. Another aPKC inhibitor, CRT0066854—a thienol[2,3-*d*]pyrimidine-based compound, has approximately five-fold selectivity for PRKCI over PRKCZ [70]. A structure-based azaquinazoline derivative of CRT0066854, CRT0329868, shows greater than 10-fold increased IC_50_ (inhibitory concentration 50 or the concentration of the inhibitor at which kinase activity is diminished by 50%) for PRKCI, favorable pharmacokinetic properties and excellent oral bioavailability [71].

A distinct class of aPKC inhibitors include the PIF-pocket targeted allosteric inhibitors. Lopez-Garcia demonstrated that the C1 domain, in the absence of PS, was still able to inhibit PRKCZ activity. The present study concluded that the C1 domain allosterically inhibited PRKCZ kinase activity [40]. In active AGC kinases, the hydrophobic motif binds to a specific pocket defined by α-B and α-C helices and β-4 and β-5 strands [40]. The C1 domain of aPKCs interacts with the catalytic domain to inhibit aPKC kinase activity. This C1 interaction has allostery with the PIF-pocket. PIF-pocket interactions could therefore regulate aPKCs independent of PS and molecules targeted to the PIF-pocket can function as specific aPKC inhibitors [40]. An inhibitor series of 4-benzimidazoyl-3-phenylbutanoic acids which have selectivity for PRKCZ over PRKCI belong to this class [72]. Other PIF-pocket allosteric inhibitors of PRKCZ includes two compounds PS168 and PS171 [40]. These compounds had five- and two-fold selectively for PRKCZ over PRKCI, respectively [40].

## aPKC expression in the nervous system

Both *Prkcz* and *Prkci* are expressed in the central nervous system (CNS) in mammals. Among the *Prkcz* transcripts, PKMζ is preferentially expressed in the brain and is not found outside the nervous system [7]. The molecular mechanism that restricts PKMζ expression to the nervous system remains uncharacterized, but it is likely that epigenetic mechanisms influence the start site choice for the *Prkcz* gene [73].

aPKC expression in the brain is also spatially distinct [74]. For example, *in situ* hybridization studies indicated that *Prkci* expression can be detected in layers II through VI of the cortex, with the highest expression detected in the cingulate, motor somatosensory and piriform cortices [74]. The olfactory tract, the CA1 (hippocampal region *cornu ammonis* 1 or the horn of Ammon 1) and CA3 (hippocampal region *cornu ammonis* 1 or the horn of Ammon 3) regions of the hippocampus and the dentate gyrus also showed high expression. *Prkci* expression was weak in the insular, thalamic and hypothalamic regions [74]. *In situ* hybridization readily detects neuronal cell-body associated RNA, but the aPKC proteins are distributed to the axonal and dendritic processes and thus may be present in additional brain regions. Immunohistochemical detection indicated prominent PRKCI staining in the white matter tracts, including corpus callosum. Immunohistochemistry, in contrast with *in situ* hybridization, detected PRKCI expression in the thalamus [75]. In the hippocampus, staining was seen in the alveus and adjacent white matter. The protein was also present in the CA1 pyramidal and dentate cell body layers [75]. PRKCI was also detected in the cerebellar cortex. PRKCI was moderately expressed in the Purkinje cells and localized to the cell body cytosol [75]. Immunoblotting also extended studies on PRKCI expression to the adult human brain where PRKCI was detected in hippocampus, caudate nucleus, superior temporal cortex and cerebellum [76].

*In situ* hybridization detected PKMζ expression in the cortical areas of the frontal forebrain, including layers II, III, V and VI, although PKMζ was absent from layers I and IV [74]. Strong expression was observed in the piriform cortex as well as the hippocampus [74,77]. PKMζ was expressed in the nucleus accumbens, olfactory tubercle and ventral palladium, but not in corpus callosum [74]. In the diencephalon, PKMζ was detected in limbic areas and the thalamus, although thalamic expression was weak (excluding the paraventricular nucleus and nucleus reuniens) [74]. The cerebellum also exhibited strong PKMζ expression in all pyramidal, granular and multiform layers [74]. Although immunohistochemistry approaches could not discriminate between PKMζ and PRKCZ in the absence of a PKMζ-specific antibody, staining with PKMζ/PRKCZ antibodies was observed extensively in the brain [75]. Intense staining was detected in the frontal and occipital cortex [75]. In the hippocampus, PKMζ/PRKCZ staining was observed in the CA1, subiculum and CA3 [75]. CA1 pyramidal and granule cell body layers stained prominently with PKMζ/PRKCZ antibodies, and this staining was relatively weaker in the CA3 cell body layer [75]. Staining was detected in the hypothalamus, and reduced staining was observed in the cerebellum and the medulla [75]. PKMζ/PRKCZ staining was seen in the molecular and granule cell layers [75]. Purkinje cell bodies were positive for PKMζ/PRKCZ stain [75]. Interesting, PKMζ expression in the brain, as validated by immunoblotting, corresponds to all brain regions tested, which were hippocampus, caudate nucleus, superior temporal cortex and cerebellum [76].

As described above, very little full-length *Prkcz* was detected in the forebrain in the *in situ* study, with a possible exception being the lateral olfactory tract [74]. However, the cerebellum had relatively high *Prkcz* expression, especially in the inner granular and Purkinje cell layers [74]. High *Prkcz* expression was described in the vestibular and pontine nuclei, inferior cerebellar peduncle, spinal trigeminal tract and the inferior olive [74]. Expression analyses using immunoblotting in the adult human brain detected PRKCZ expression only in the cerebellum and not in the hippocampus, caudate nucleus or superior temporal cortex [76]. The functional consequences of this spatial distribution of the aPKC proteins in the brain remain unknown.

There also appears to be diversification of aPKC expression within the cell types of the brain. For example, full-length PRKCI is detected in neurons, astrocytes and oligodendrocytes [www.brainrnaseq.org]. However, PKMζ is expressed in neurons and oligodendrocytes, but not in astrocytes [www.brainrnaseq.org]. Since radial glial cells sequentially give rise to neurons, astrocytes and oligodendrocytes, it will be important to study the regulation of aPKC during neural stem cell differentiation and its functions in various neural cell types.

## aPKC, polarity and neuronal differentiation

**Figure 4:**
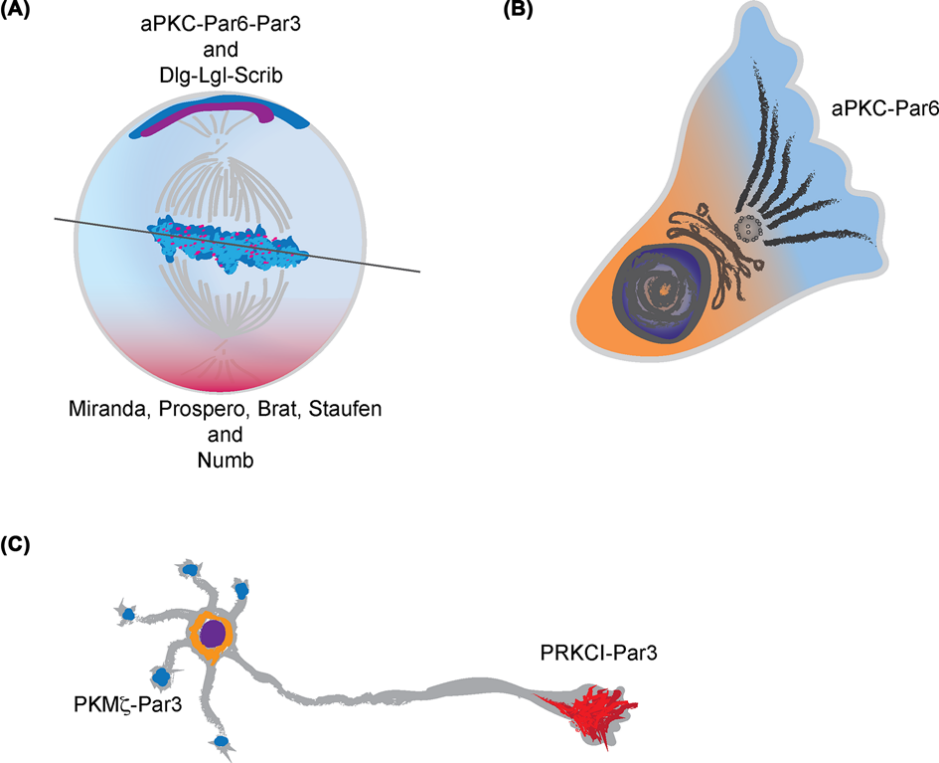
aPKC function in neural differentiation, migration and maturation Schematics of aPKC-containing complexes and the regulation of (**A**) asymmetric cell division of *Drosophila* neuroblasts, (**B**) astrocyte migration in culture and (**C**) axon-dendrite specification in newborn neurons. During neuroblast division, the cortical aPKC-complex regulates basal determinant positioning and spindle orientation. During the migration of primary astrocytes isolated from 17-day-old rats in culture, the aPKC-complex is involved in polarizing the Golgi apparatus, microtubule organizing center and the cytoskeleton toward the leading edge of the cell as well as in regulating directional migration of the cell. Differential aPKC-complexes either favor or oppose axon-specification. PRKCI-Par3 favors axon specification. PKMζ-Par3 opposes axon specification. These polar forces result in the specification of a single neurite, out of many, into an axon.

In flies, the asymmetric division of the neuroblast results in differentiation, while symmetric divisions are synonymous with self-renewal [78]. During asymmetric division, neuroblasts polarize to form distinct cortical domains (Figure 4A). This polarization restricts Miranda, Prospero, Brat, Staufen and Numb, proteins of the basal complex, to the ganglion mother cell (GMC) after neuroblast division. Phosphorylation of Miranda and Numb is critical for their asymmetric localization. GMC divides once, generating two post-mitotic neurons or glial cells. The restriction of the basal complex to the GMC requires the activity of the apical complex, formed by Bazooka, DaPKC and Par6. Bazooka, DaPKC and Par6 are localized to the apical cortex, with aPKC functioning as the effector that drives phosphorylation of Miranda and Numb. In *daPKC* loss-of-function mutants, fewer neuroblasts were observed in comparison with wild-type [79,80]. In contrast, a DaPKC-CAAX (C: cysteine, A: aliphatic amino acid, A: aliphatic amino acid and X: any amino acid) construct enhanced symmetric divisions and self-renewal, thereby increasing the number of neuroblasts [80]. The DaPKC-CAAX construct is DaPKC tagged with the tetrapeptide CAAX motif commonly found in Ras and Ral GTPases. This CAAX consensus sequence is a signal for a series of post-translational modifications including prenylation, which directs intracellular trafficking and translocation to the plasma membrane. DaPKC-CAAX was membrane-localized and therefore active. Furthermore, it distributed indiscriminately throughout the entire cortex. Therefore, this construct was effectively a gain-of-function mutation due to activation coupled with loss of its apical restriction. Thus, the exquisite regulation of spatial (apical) aPKC activity in the cortex is critical for the establishment of neural stem cell *versus* progenitor identity, in part through the asymmetric segregation of Numb [81]. Another study generated an analog-sensitive mutant of DaPKC [82]. Analog-sensitive mutants substitute a structurally conserved bulky amino acid such as methionine, leucine, phenylalanine or threonine in the active site of kinases termed as ‘gatekeeper residue’ to a residue with a much smaller side chain [83]. This enables binding of kinase inhibitors with large bulky groups, such as C3-tolyl ring, specifically to the engineered kinase while sparing wild-type kinases [83]. Employing the analog-sensitive DaPKC mutants, the authors were able to demonstrate that acute inhibition of DaPKC during neuroblast polarity establishment abolished the asymmetric localization of Miranda, as well as Numb, while PARD3 and DLG (discs large) were mostly unaffected [82].

Similar functional roles of aPKC in the regulation of symmetric *versus* asymmetric cell divisions of neuroblasts have been described in some vertebrates. In Zebrafish (*Danio rerio*), the *heart and soul* (*has*) mutation leads to loss of *Prkci* function. In *has* mutant embryos, neuroepithelial cells have been reported to switch from planar to oblique divisions [84]. The plane of cell division in the neuroepithelium has been associated with cell fate (symmetric division and proliferation *versus* asymmetric division and differentiation) in vertebrates. Correspondingly, the neuroepithelial precursor population is decreased in *has* mutants with a concomitant increase in oligodendrocyte progenitor cells [84]. Interestingly, the number of newborn neurons in the spinal cord was not affected in this mutant [84]. Similarly, the number of motor and interneurons in chick embryos was not affected after overexpression of an active, mislocalized PRKCZ [85].

During neurogenesis in the African clawed frog (*Xenopus laevis*), apical-basal polarized superficial cells retain their progenitor fate while non-polar deep cells differentiate into neurons. aPKC-CAAX suppresses neurogenesis and promotes cell proliferation [86]. However, the mechanism by which aPKC regulates proliferation *versus* differentiation appears to be through the regulation of cell cycle phase length rather than through regulation of cell polarity. Phase length within the cell cycle, *viz.* G_1_ (gap phase 1 of the cell cycle) phase length, has been associated with the decision of proliferative *versus* differentiation divisions. For example, during mouse cortical development, a shorter G_1_ phase is associated with proliferative divisions while a longer G_1_ phase is associated with differentiation and neurogenesis [87,88]. In *Xenopus* neuroepithelial cells, p27Xic1 functions as a Cdk2 (cyclin-dependent kinase 1) inhibitor and promotes neuronal differentiation. aPKC phosphorylates p27Xic1, which interferes with its inhibitory effect on Cdk2. This results in shortened G_1_- and S-phases and the promotion of neural progenitor proliferation [89].

In *in vitro* experiments, PRKCI expression favors neuronal differentiation of PC12 (pheochromocytoma 12 cell line) cells after NGF (nerve growth factor) treatment [23]. Other experiments suggested that PRKCZ binds a neuronal differentiation factor, TRIM32, and retains it in the cytoplasm, thus preventing differentiation of mouse neural stem cell cultures [90]. During induced differentiation, PRKCZ amounts are down-regulated and TRIM32 translocates to the nucleus. Studies using aPKC-specific shRNAs (short-hairpin RNAs) have reported direct roles of both aPKCs in mammalian neuronal development. Embryonic day 12 (E12) nestin-positive neural precursor cells require the function of CREB-binding protein (CBP), a histone acetyltransferase, for differentiation [91]. The enzymatic activity of CBP is dependent on PRKCZ phosphorylation. Thus, aPKC-driven phosphorylation of CBP functions as an epigenetic switch to promote the differentiation of neural precursors, especially into astrocytes and oligodendrocytes. Neuronal differentiation was also affected in the absence of this phosphorylation event [91]. The phosphorylation-deficient mutant of CBP showed impaired hippocampal neurogenesis and fear-memory deficits [92] (please see ‘**aPKC and neuronal function**’ section). PRKCI, in contrast, was reported to be required for the maintenance of Pax6 and Sox2-positive radial precursors [93]. An independent study by Tischfield et al. [94] used ZIP to investigate aPKC function during *in vitro* differentiation of specific neuronal subsets from mouse embryonic stem cells. Almost half of the inhibitory GABAergic (GABA, γ aminobutyric acid) interneurons in mice and humans are born within the medial ganglionic eminence (MGE) of the subcortical telencephalon. These neurons become either parvalbumin (Pv)-positive or somatostatin-positive (Sst). Tischfield et al. [94] reported that ZIP shifts MGE neurogenesis toward symmetric, intermediate progenitor divisions and towards the Pv subtype.

In contrast to the silencing studies, genetic knockout studies of aPKC in mice do not result in changes in neural stem cell proliferation or differentiation. *Prkci* is an essential gene for viability, as embryos lacking it do not develop past E9 [95]. Therefore, to address its function in neural stem cell proliferation or neuronal differentiation, a neural precursor-specific *Prkci* knockout was generated using *Nestin*-Cre. These experiments revealed that mice with homozygous deletion of this gene in neural tissue were born in an expected Mendelian ratio, as were mice with wild-type or heterozygous *Prkci* alleles [96]. However, growth retardation was noted in the homozygous deletions by five days after birth and all neural-tissue specific *Prkci* deleted mice died within a month after birth. Using reporters, it was determined that this Cre recombinase (Cre is derived from ‘causes recombination/cyclization’) was active at E13.5 and loss of PRKCI protein occurred by E15.5. *Prkci* deletion led to defects in neuroepithelial cell adhesion and packing within the neuroepithelium, with indistinguishable and disorganized ventricular and subventricular zones. At a subcellular level, neuroepithelial adherens junctions were disrupted in neural-tissue-specific *Prkci-*deleted mice. Importantly, neuronal differentiation was not affected [96]. In contrast with PRKCI, *Prkcz* deleted mice had grossly normal brain development and anatomy [97,98]. The reasons why genetic deletion *versus* gene silencing of aPKCs in mice resulted in completely different findings remain unknown. It is possible that genetic knockdown, especially when embryonic, results in up-regulation of alternative compensatory mechanisms. A more acute knockdown with shRNA may avoid this issue and therefore, more successfully reveal the gene’s function. Alternatively, the shRNAs used might have some non-specific effects and aPKCs do not function in neural stem cell proliferation or differentiation.

## aPKC and neural migration

aPKC is an important regulator of cell polarity and directional migration. In rat astrocytes, the microtubule organizing center (MTOC), the microtubule cytoskeleton, as well as the Golgi apparatus polarize towards the leading edge during directional migration (Figure 4B). PRKCZ was reported to enable this polarization by regulating glycogen synthase kinase 3β (GSK3β) activity [99,100]. As discussed before, full-length PRKCZ is not detected in mouse astrocytes and therefore unlikely to be expressed in rat astrocytes. It is more likely that this is a case of mistaken identity due to the cross-reactivity of the antibody used, as well as that of ZIP, and as overexpressed PRKCZ can have overlapping functions with PRKCI. The aPKC involved should be PRKCI and not PRKCZ. PRKCI also appears to regulate the polarization of adenomatous polyposis coli protein (APC) and DLG1 during this process [101].

During the development of the cerebral cortex, postmitotic neurons migrate towards their final destination to establish the cortical layers. Organotypic slices of E12.5 or E13.5 mice brains, cultured for two days *in vitro*, recapitulated aspects of cortical development. Treatment of organotypic cultures with a high concentration of pan-PKC inhibitor BIM1 and Ro318220, but not the classical/conventional PKC-specific inhibitor Gö6976, resulted in migration defects [102]. Lower concentrations of BIM1 that inhibit classical/conventional and novel, but not atypical, PKCs also failed to disrupt *in vitro* development. Based on these results it was interpreted that aPKCs drive neuronal cell migration during cortical morphogenesis [102]. An unbiased chemical screening approach using this organotypic slice culture system and the National Cancer Institute (NCI) Diversity Set representing 140,000 compounds identified 11 compounds that disrupted neuronal migration. One of the compounds, 17-(2-Aminothiazol-4-yl)-11-hydroxy-10,13-dimethyl-1,7,8,10,11,12,13,15,16,17-decahydro-2Hcyclopenta[a]phenanthren-3(6H,9H,14H)-one, salt with 4-bromobenzensulfonic acid, reduced the activation loop phosphorylation of aPKCs [103]. Live imaging of cerebellar granule cells migrating on astroglial fibers revealed that the aPKC-binding-protein PARD6 localized to the centrosome of the granule cells [104]. The centrosome moves forward and then pulls the nucleus in an orchestrated manner for neuronal migration. Importantly, overexpression of PARD6A disrupted this movement pattern and impaired glial-guided neuronal migration [104].

Apart from radial glia-guided neuronal migration, newborn neurons use somal translocation, as well as multipolar migration, to relocate during development. In somal translocation, neurons extend long apical and basal processes before shifting the position of the nucleus. In multipolar migration, the cell extends many short dynamic processes and crawls towards its final destination. Retinal ganglion cells in the larval zebrafish retina were able to use the less efficient multipolar migration mode when somal translocation was interfered with by chemical or genetic means. Overexpression of PRKCZ-CAAX abolished both forms of migration, suggesting a critical function of this kinase in neuronal migration *in vivo* [105]. These results remain to be reconciled with the phenotypes observed with the genetic knockouts.

## aPKC and neuronal maturation

One of the more striking functions ascribed to aPKC in neurons is that of axon specification. The first clues to aPKC function in axon specification came from studies using ZIP in newborn mammalian hippocampal neurons in culture. It was inferred that PRKCZ kinase activity enabled axon specification [106]. However, mammalian hippocampal neurons appear to express only PRKCI and PKMζ, not PRKCZ. Subsequently, it was demonstrated that PRKCI favored axon determination while PKMζ inhibited this process [53] (Figure 4C). Both aPKCs bind PARD3, and the competition between them might ensure the specification of a single neurite into an axon [31,53,107]. A function of PRKCZ in axon-dendrite specification in the enteric nervous system (ENS) was also reported [108]. ZIP treatment increased the percentage of neurons without axons or neurons with supernumerary axons over control treatment when ENS precursors isolated from E14.5 rat embryos were cultured for 48 h [108]. Inhibition of the aPKCs also reduced the rate of neural crest migration into the distal bowel in organotypic cultures of mouse E11.5 gut [108]. None of these studies have been validated using genetic knockouts, however.

Many upstream activators and downstream effectors of aPKC in *in vitro* axon-dendrite formation have been identified. Upstream activators include Dvl (dishevelled), a component of the Wnt signaling pathway, Nup358, a nucleoporin, and receptor tyrosine kinases (RTKs) such as IGF1-R (insulin-like growth factor 1-receptor) or EGFR (epidermal growth factor receptor) [28–30,106]. Wnt-frizzled signaling also directs spinal cord commissural axon turning and midline crossing. ZIP treatment randomized anterior-posterior positioning of commissural neurons in open-book rat spinal cord explant cultures [109]. Many downstream effector molecules have also been described, including GSK3β and MARK2 (microtubule-affinity-regulating-kinase 2) [100,110]. A more extensive discussion on this topic can be found in Hapak et al. [31].

In contrast, *in vivo* studies have failed to specify a function of aPKCs in axon-dendrite formation. Studies on mushroom body neurons from *DaPKC-*null *Drosophila* third instar larva using the MARCM (mosaic analysis with a repressible cell marker) system for GFP (green fluorescence protein)-labeling failed to reveal any defects in dendrite or axon morphology. Even in adult flies, the α′β′ and αβ neurons showed normal axon projections, although the number of these neurons were reduced due to neuroblast differentiation defects [111]. A neural cell-specific genetic ablation of *Prkci* (at E15.5) in mouse or the germline deletion of *Prkcz* in mouse also did not affect axon specification *in vivo* [96–98]. Additionally, the use of *Camk2*-Cre or *Synapsin*-Cre to ablate *Prkci* failed to induce neuronal developmental defects [112]. Whether the fact that recombination occurs only approximately 8–16 weeks with the *Camk2*-Cre or *Synapsin*-Cre precludes effects on axon-dendrite specification *in vivo* remains unknown. However, it appears that aPKC function, if any, may be redundant for axon specification and growth *in vivo*.

## aPKC and neuronal function

**Figure 5:**
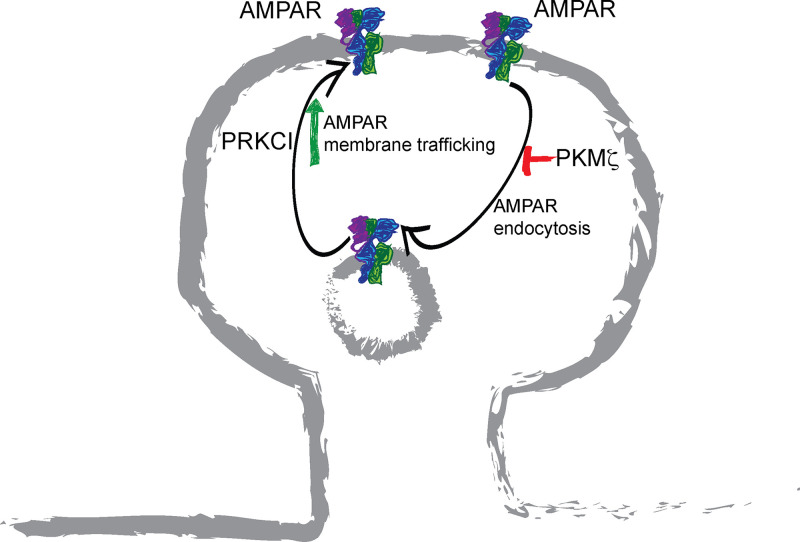
aPKCs and AMPAR trafficking Both PRKCI and PKMζ are postulated to play a role in learning and memory by enhancing membrane-localized AMPAR. While PRKCI can enhance trafficking of internal AMPAR pools to the synapse membrane, PKMζ prevents internalization of membrane-associated pools. These proteins can compensate for each other during learning and memory formation.

A holy grail in neuroscience is the quest for the molecular substrate of learning and memory. The nervous system-specific aPKC - PKMζ - appears to fit the characteristics of the elusive memory molecule. Protein kinases, including CaMKII and PKCs are known to mediate LTP. The induction of LTP is associated with Ca^2+^ influx. However, maintenance would require persistent activation of kinases [113,114]. The constitutive activity of PKMζ makes it an attractive candidate. PKMζ concentrations in the cytosol of hippocampal tissue were found to be elevated during the maintenance phase 30 min after tetanization [115]. Furthermore, the degree of long-term excitatory postsynaptic potential (EPSP), but not short-term potentiation, correlated with increased amounts of PKMζ [116]. In a landmark paper in 2002, Sacktor’s laboratory [59] demonstrated that PKMζ is sufficient and necessary for LTP maintenance. The authors used purified PKMζ in electrodes used for whole-cell recording of long-term excitatory postsynaptic currents (EPSCs) to demonstrate that PKMζ enhances EPSCs. Furthermore, they used ZIP and chelerythrine as PKMζ inhibitors 1–5 h after tetanization to demonstrate that this kinase is necessary for LTP maintenance. In fact, PKMζ only functions at late phases of LTP and not at initiation [117]. 2006 marked the first *in vivo* investigation of the function of PKMζ in learning and memory in mammals. By injecting ZIP into rat hippocampus, Pastalkova et al. [118] documented the reversal of one-day-old spatial memory in active place avoidance tests. This was extended to long-term associative memory. Conditional taste aversion was erased by ZIP infusion to the insular cortex, even when injected 25 days after training [119]. Consistent with the above-mentioned studies, viral overexpression of PKMζ in the rat insular cortex enhanced long-term memory [120]. The amount of PKMζ in the dorsal hippocampus persists for up to one month after training in spatial conditioning trials [121]. Inhibiting this increase by injecting anti-sense oligonucleotides into the hippocampus prevented long-term memory formation [121]. PKMζ function in memory was extended to various other paradigms by independent laboratories [122], although some studies observed regional/neuronal subtype-specificities with respect to the role of PKMζ [123,124]. One such interesting study demonstrated that administration of ZIP in the nuclear accumbens inhibited the retrieval of drug (remifentanil)-associated memory and drug seeking behavior [125]. Molecularly, PKMζ functions by inhibiting AMPA (α-amino-3-hydroxy-5-methyl-4-isoxazolepropionic acid) receptor internalization and increasing its concentration at postsynaptic sites [126] (Figure 5). Further details on PKMζ function in learning and memory can be found in a series of excellent reviews by Sacktor [33,127,128].

Meanwhile, independent studies in *Aplysia* provided additional support for the PKMζ paradigm. A PKMζ analog, produced by cleavage of the *Aplysia* aPKC, PKC Apl III, was identified by Bougie et al. [22]. Intrahemocoel injection of ZIP or chelerythrine erased seven-day-old memories as tested by siphon-withdrawal reflex after sensitization [129]. Furthermore, serotonin was reported to induce calpain-dependent and protein-synthesis-dependent cleavage of PKC Apl III into its PKM form [124]. Serotonin induced long-term memory through the formation of new synapses, and the addition of new synapses could be reversed by chelerythrine [130]. Similar studies in *Drosophila* revealed the presence of a PKMζ analog, DaPKM [131]. Expression of mouse PKMζ or DaPKM enhanced memory in Pavlovian olfactory learning tasks in flies [131]. aPKC, including truncated PKMζ−like proteins, have been reported to play a role in memory in land snails *Helix lucorum* [132]. aPKC expression was increased in this species following taste aversion learning, but not after contextual fear conditioning [132].

Amidst the excitement regarding the function of PKMζ in learning and memory, concerns were being raised by some investigators. John Lisman questioned whether ZIP was acting solely via PKMζ or other targets were involved [133]. The suitability of ZIP and chelerythrine as specific PKMζ inhibitors was brought into question by Wu-Zhang et al. [56,57]. This was debated, and the concentration and effectiveness of ZIP re-validated by Sacktor and Fenton [134] and by Yao et al. [58]. The labile nature of PKMζ [45,135] was presented as a paradox for the persistence of its kinase activity [135]. As discussed earlier, the feedback loop wherein PKMζ inactivates the translation inhibitor Pin1 by phosphorylation, leading to increased translation of this protein can be invoked to explain the persistence of long-term memory. In fact, a computational model that involves interactions between two coupled feedback loops – that of PKMζ-dependent Pin1 inactivation and increased PKMζ protein translation and that of PKMζ-dependent insertion of AMPAR at the synapse and the mutual dependence for maintenance of PKMζ and AMPAR at the synapse – recapitulates a bistable mechanism for synaptic potentiation [136].

Two back-to-back papers published in 2012 using *Prkcz* knockout mice further challenged the role of PKMζ by demonstrating that the genetic ablation of *Prkcz* was dispensable for learning and memory [97,98]. Mostly mice with a germline deletion of this gene were used in these studies, and they failed to reveal the expected learning- and memory-deficient phenotype predicted based on ZIP experiments [97,98]. Even the inducible deletion of *Prkcz* using the CaMKII-CreER^T2^ system in 8–10-week-old adult mice failed to show any difference in LTP when compared with control wild-type mice, suggesting that the lack of phenotype in the germline deletion mice was not due to unknown compensatory mechanisms up-regulated in the long-term absence of PKMζ [98]. Importantly, in both studies, ZIP reversed memory in *Prkcz* knockout mice [97,98].

These results suggest that ZIP can function independent of its action on PKMζ. One study indicated that ZIP inhibits neural activity comparable in magnitude, but slower in onset and longer lasting than the sodium channel blocker lidocaine [70]. Another study demonstrated that ZIP, as well as the negative control scrambled ZIP, enhanced spontaneous activity, sustained elevation of intracellular Ca^2+^ and even excitotoxic death at 5–10 μM concentrations in a study using dissociated rat hippocampal neurons in culture [137]. A study in *Aplysia* suggested that the antimnemonic actions of chelerythrine involved the induction of epigenetic changes, and residual long-term memory could persist following reversal of new synapse addition [130]. Another *Aplysia* study demonstrated that both ZIP and chelerythrine inhibit the classical/conventional PKC Apl I, as well as the aPKC Apl III [47]. Moreover, depolarization of sensory neurons with 100 mM KCl (potassium chloride) alone or KCl plus 5-HT (5-hydroxytryptamine i.e. serotonin) or evoked action potential activity resulted in cleavage of Apl I, but not Apl III [47]. Consistent with a role of the PKM form of the *Aplysia* classical/conventional PKC Apl I and not that of the *Aplysia* aPKC Apl III, dominant-negative PKM from Apl I, but not from Apl III, blocked activity-dependent intermediate-term facilitation, a form of synaptic plasticity and memory, in sensory neurons [47]. These results suggest that ZIP does not function solely through inhibition of the PKM form of aPKC Apl III, at least in *Aplysia*.

The publication of these papers generated debate on the authenticity of PKMζ as the memory molecule and attempts were made to understand the divergent findings [58,123,138–141]. Sacktor’s laboratory reported that, in the absence of *Prkcz*, PRKCI amounts increase and PRKCI can compensate for PKMζ function in LTP maintenance [41]. An independent study had previously identified PRKCI as an important regulator of LTP [142]. Using shRNA to silence PRKCI, these authors discovered that LTP was reduced in its absence [142]. An shRNA-resistant mutant of *Prkci* rescued this defect. Conversely, active PRKCI enhanced EPSCs [142]. It was concluded that PRKCI functioned earlier, just after LTP induction, while PKMζ functioned in late-LTP. Interestingly, in a separate study, this group used CaMKII-Cre to generate a neuron-specific deletion of *Prkci* and found that these animals fail to phenocopy the shRNA phenotype [143]. In this case, PKMζ expression was enhanced during LTP, associative learning tasks (such as trace auditory fear conditioning and contextual fear conditioning) and during spatial learning, but was not enhanced basally [143]. Furthermore, PKMζ compensated for PRKCI function in LTP. However, if the task was made difficult by reducing the number of training trials, an LTP defect was observed in mice lacking neuronal PRKCI [143]. PRKCI, similar to PKMζ, enhances AMPA receptor concentration at the postsynaptic sites. Unlike PKMζ, which maintains the GluR2 (glutamate receptor 2) subunit at the synapse thereby preventing AMPA receptor internalization, PRKCI is required for the phosphorylation of the glutamate receptor 1 (GluR1) subunit and the recruitment of AMPA receptors to the synapse [126,142] (Figure 5). The use of PRKCI-specific inhibitor 5-amino-1-(1R,2S,3S,4R)-2,3-dihydroxy-4-methylcyclopentyl)-1H-imidazole-4-carboxamide, which does not inhibit PRKCZ, confirmed that this reagent could reverse established LTP in *Prkcz*^−^/^−^brain slices, but not in wild-type brain slices [144]. However, if included before tetanization, the PRKCI inhibitor could block the induction of early-LTP [144]. Thus, in the absence of either *Prkcz* or *Prkci*, the remaining aPKC is induced in a learning-dependent manner and compensates for the loss. Although mice with neuron-specific ablation of both *Prkcz* and *Prkci* have not been tested so far, a scenario where PKMζ and PRKCI compensate for each other in memory function is highly plausible since both these kinases are inhibited by ZIP. Nonetheless, PKMζ and PRKCI may not be simply redundant backups for learning and memory functions. Each kinase, while capable of compensating for the other, may be adapted to specific types of memory in wild-type animals. Interestingly, in *Aplysia*, different PKMs function in the maintenance of specific memories in discrete neuronal subsets. While PKM Apl I, PKM Apl II (*Aplysia* novel PKC) and PKM Apl III are involved in non-associative long-term facilitation (LTF) in sensory neurons, PKM Apl II and PKM Apl III function in associative LTF in gill motor neuron L7 and PKM Apl II regulates associative LTF in sensory neurons [145].

## aPKC in regeneration

In the CNS, damaged axons are prevented from regenerating due in part to the lack of cell-intrinsic growth signals and also to growth-inhibitory environmental signals such as myelin, myelin-associated proteins and chondroitin sulfate proteoglycans (CSPGs). Functionally antagonistic signaling pairs are attractive candidates for symmetry breaking and for spurring axon regrowth after axonal injury and degeneration [107]. In this regard, PRKCI and PKMζ might function as a Turing reaction-diffusion system for symmetry breaking in axotomized neurons for axon specification and re-growth [107]. Therefore, despite the failure to recapitulate the axon growth-regulatory effects of aPKC *in vivo* during development, we postulate that these kinases may yet be functionally important in the context of CNS regeneration. Consistent with this idea, NG2 (neural/glial antigen 2) CSPG is known to activate PRKCZ, and ZIP treatment reverses the growth inhibition induced by NG2 CSPG in cultures of dissociated postnatal rat cerebellar granule neurons or adult mouse dorsal root ganglia neurons [146]. All three aPKCs are expressed in ventral spinal cord segments (C3-C5), including in the phrenic neurons [147]. Following C2 cervical hemisection, total PRKCI/PRKCZ expression was unchanged but the expression and activity of PKMζ was increased in phrenic neurons [148]. These results suggest that PRKCI and PKMζ might function as an antagonistic pair during functional recovery following spinal cord injury.

## Neuronal aPKCs in human diseases and disease models

A number of association studies in human diseases have suggested intriguing connections between aPKCs and psychiatric diseases. When Iwamoto and colleagues correlated single nucleotide polymorphisms (SNPs) with gene expression changes in human prefrontal cortex, a CC genotype at a SNP was associated with the highest expression of the *PRKCI* transcript, followed by TC and then TT [149]. Interestingly, the SNP identified in *PRKCI* in this study was the same as that identified by The Wellcome Trust Case Control Consortium association study as significantly associated with bipolar disorder [150]. Another independent study also suggested an association between *PRKCI* and bipolar and alcohol use disorders [151]. The drug topiramate has been used in epilepsy, migraines, alcohol-dependence, bipolar disorders and methamphetamine-dependence. In a study examining genome-wide transcriptional changes in whole blood from topiramate-responders (patients who stopped using methamphetamine) *versus* non-responders (patients continuing to use methamphetamine) within a cohort of methamphetamine-dependent individuals, *PRKCI* was identified as a topiramate target gene. Increased *PRKCI* expression was seen in patients who stopped using methamphetamine after 8 weeks on topiramate [152]. Another example demonstrating a potential association between aPKCs and mental health was in a study looking at *PRKCI* expression in suicide victims. In it, the authors found that the expression level of *PRKCI* in the pre-frontal cortex was increased in suicide victims as compared with non-suicide controls [153]. Interestingly, the incidence of bipolar disorder was higher in people who did not commit suicide, whereas the incidence of major depressive disorder was higher in the suicide group. *PRKCZ* was also identified as a susceptibility locus for bipolar disorder in a genome-wide association study (GWAS) of 600 patients and 605 controls [154]. The SNPs mapped to the promoter region of the shorter transcripts within *PRKCZ*, suggesting that it is PKMζ that is likely involved in this disease. Analyses of copy number variants (CNVs) data from the International Schizophrenia Consortium (ISC) in 2008 identified 34 genomic deletions and 91 duplications in 3,391 schizophrenic patients compared with 17 deletions and 58 duplications in 3,181 controls. It was suggested that *PRKCZ* might be a gene of interest, although the rarity of disease-specific CNVs precluded confirmed associations [155]. In the topiramate study discussed above, *PRKCZ* was a transcript down-regulated at 8 weeks in topiramate-responders [152]. Despite these tantalizing associations, whether aPKCs are functionally involved in complex, higher cortical activities in humans remain uncharacterized. The expression of aPKCs in the brain may be under complex regulation, making single-time-point association studies difficult to interpret. In a North American population, sunlight exposure was associated with CpG (cytosine poly guanine sequence where the cytosine can be methylated) methylation of a site located in *PRKCZ* [156], indicating that seasonal or geographical environmental factors may affect the expression of this kinase.

aPKCs may be associated with neurodevelopmental and neurodegenerative diseases. Human *PRKCZ* is located on chromosome 1p36 (band 36 of the short arm [p] of chromosome 1). Patients with monosomy 1p36 syndrome exhibit clinical features of intellectual disability, epilepsy and craniofacial abnormalities [157]. Whether any of these can be causally linked to *PRKCZ* remain undetermined. The association appears more definitive in multiple sclerosis. Examination of pathology-free regions of brains from patients who suffered from multiple sclerosis *versus* control brains revealed hypermethylation at the *PRKCZ* locus in the diseased brain [158].

Alzheimer’s disease (AD) is a progressive neurodegenerative disease that leads to irreversible memory loss and severe cognitive impairments. PKMζ was observed to aggregate in neurofibrillary tangles (NFTs) along with tau [76]. However, unlike tau or PRKCI that were found to accumulate in all areas of the brain, PKMζ-containing NFTs were observed specifically in the limbic system or the medial-temporal lobe structures including the hippocampus, entorhinal cortex, subiculum and the amygdala and not outside these areas [76]. Although not associated with any specific brain region, PRKCI immunostaining was observed not only in AD, but also in Pick bodies of hippocampal dentate gyrus neurons and neocortical neurons in Pick Disease patients, globose triangles and tufted astrocytes in subthalamic, mesencephalic and cerebellar dendate nuclei, as well as in the inferior olive in Progressive Supranuclear Palsy patients, and tau-positive astrocytic inclusions in the cerebral cortex, and neuronal and glial inclusions in the basal ganglia in patients with Corticobasal degeneration [159]. PRKCI also stains Lewy bodies in α-synucleinopathies such as Parkinson’s Disease and dementia with Lewy bodies [159].

Apart from the association studies in humans, various mouse models of diseases have also suggested a role of neuronal aPKCs. In a superoxide dismutase 1 (SOD1) (G93A) mouse model of amyotrophic lateral sclerosis (ALS), Tury et al. [160] observed that aPKC expression was increased in motor neurons of the lumbar spinal cord and accumulated in extracellular aggregates in increasing amounts with disease progression. Furthermore, neuronal PRKCI activation by insulin enhanced Aβ_1-40/42_ amounts and tau-phosphorylation, suggesting that this kinase might mediate aspects of AD pathology [161]. In a study of diet-induced obesity, the genetic ablation of *Prkci* in proopiomelanocortin (POMC) neurons disrupted leptin action and increased glucose intolerance, insulin resistance and obesity in male mice fed high-fat diets [162].

Transcriptional profiling after ethidium-bromide induced focal demyelination followed by remyelination in rat CNS identified *Prkcz* as an up-regulated gene that also correlated with RXR (retinoid X receptor) pathway activation, oligodendrocyte differentiation and remyelination [163]. Polarity genes such as *Pard3* and *Scribble* (*Scrib*) have been implicated in myelination/remyelination in mouse models [164–168]. Sirtuin 2 (SIRT2) deacetylates PARD3, which in turn decreases aPKC activity. Mice overexpressing or lacking *Sirt2* in Schwann cells displayed delayed myelination [169].

A number of papers (reviewed in [170]) have implicated PKMζ in chronic pain models. Most of the studies employed ZIP. In a study by Nasir et al. ZIP did not affect mechanical or thermal pain-sensitivity in inflammatory or neuropathic pain models in rats [171]. However, late onset contralateral allodynia was markedly reduced by ZIP. Similarly, *Prkcz*^−^/^−^ but not wild-type mice had reduced contralateral allodynia in the neuropathic pain model [171]. Mild but not moderate formalin-induced pain, long-lasting referred pain associated with visceral injuries and referred pain following muscle injury were also alleviated in rats upon intrathecal administration of ZIP [171]. Interestingly, the efficacy of PKMζ ablation/pharmacological inhibition in allodynia was restricted to male mice [171].

A role of *Prkcz* in avoiding binge drinking was also established using genetic knockouts. *Prkcz* expression positively correlated with ethanol consumption in mice [172,173]. Consistent with these reports, Lee et al. reported that *Prkcz*^−^/^−^ mice consumed more alcohol in comparison with wild-type littermates for an intermittent–access-alcohol-consumption protocol, but not in drinking-in-the-dark or 24-h-continuous-access protocols [174]. Therefore, it was speculated that a *Prkcz*-mediated negative feedback loop might suppress binge drinking.

## Conclusions

A wealth of data from human disease-association studies, genetically engineered mouse models of diseases and *in vitro* and *in vivo* studies related to aPKC expression and function points to an important function of aPKCs in neurobiology. Yet, aPKC function in the nervous system still eludes an unequivocal consensus. Many of the differences between studies on aPKC seemingly stem from the employment of gene deletion or knockout approaches *versus* gene silencing approaches. Perhaps the simultaneous genetic ablation of *Prkci* and *Prkcz* using neuron-specific Cres or even neuron-specific, inducible CreER^T2^ (Cre recombinase estrogen ligand-binding domain fusion protein that is used for inducible recombinase by tamoxifen application) might resolve this issue. Despite controversies, we suggest that aPKCs remain attractive candidates for mediating a variety of critical neuronal functions. Careful and comprehensive studies might still elucidate the fundamental principles of aPKC-driven biology, with relevance to morphogenesis, physiological function and the etiology of neurological or psychiatric diseases. Understanding aPKC function may potentially allow the development of novel therapeutic interventions in insofar untreatable human diseases.

## Abbreviations

AD: Alzheimer’s disease
AGC: protein kinase A, protein kinase G and protein kinase C
AMPA: α-amino-3-hydroxy-5-methyl-4-isoxazolepropionic acid
aPKC: atypical protein kinase C
C: spinal cord cervical vertebrae
Ca^2+^: calcium ion
CAAX: cysteine, aliphatic amino acid, aliphatic amino acid and any amino acid consensus sequence from Ras/Ral that undergoes prenylation and mediates association with cellular membrane
CaMKII: Ca^2+^/Calmodulin-dependent protein kinase
CA1: hippocampal region *cornu ammonis* 1 or the horn of Ammon 1
CA3: hippocampal region *cornu ammonis* 1 or the horn of Ammon 3
CBP: CREB-binding protein
Cdk2: cyclin-dependent kinase 1
cDNA: complementary DNA
CNS: central nervous system
CNV: copy number variant
Cre: a recombinase originally termed ‘causes recombination/cyclization recombinase’
CSPG: chondroitin sulfate proteoglycan
DAG: diacylglycerol
DaPKC: *Drosophila* aPKC
E: embryonic day
ENS: enteric nervous system
EPSC: excitatory postsynaptic current
GFP: green fluorescence protein
GluR1: glutamate receptor 1
GMC: ganglion mother cell
GSK3β: glycogen synthase kinase 3β
G_1_: gap phase 1 of the cell cycle
IGF-1: insulin-like growth factor 1
KCl: potassium chloride
LTF: long-term facilitation
LTP: long-term potentiation
MGE: medial ganglionic eminence
NCI: National Cancer Institute
P: postnatal day
PARD3: partitioning defective
PB1: Phox/Bem1
PDK1: pyruvate dehydrogenase kinase 1
Pin1: peptidylprolyl *cis/trans* isomerase, NIMA-interacting 1
PI3-K: phosphoinositide 3-kinase
PKA: protein kinase A
PKC: protein kinase C
PKC3: *Caenorhabditis elegans* aPKC
PS: pseudosubstrate sequence
Pv: parvalbumin
p62/SQSTM: p62/sequestosome-1
S: synthesis phase of cell cycle
shRNA: short-hairpin RNA
SNP: single nucleotide polymorphism
Sst: somatostatin
WNT: portmanteau from Wingless and Int
ZIP: ζ-inhibiting peptide
1p36: band 36 of the short arm [p] of chromosome 1
